# The triangular drivers of bone aging: mechanistic insights and therapeutic targets in cellular senescence, estrogen deficiency, and gut microenvironment dysregulation

**DOI:** 10.3389/fcell.2026.1727398

**Published:** 2026-03-24

**Authors:** Zhiwei Liu, Yuqian Peng, Jinyu Hou, Guodong Zhang, Jindong Wang

**Affiliations:** 1 The Second Clinical Medical College, Shanxi Medical University, Taiyuan, Shanxi, China; 2 Department of Orthopaedics and Sports Medicine, The Second Hospital of Shanxi Medical University, Taiyuan, Shanxi, China

**Keywords:** bone aging, cellular senescence, estrogen deficiency, gut microenvironment, osteoporosis

## Abstract

A critical component of organismal aging is skeletal aging, which plays a pivotal role in the onset and progression of various age-related bone disorders and imposes a substantial burden on global public health. Recent studies have increasingly demonstrated that cellular senescence, estrogen deficiency, and gut microenvironment dysregulation serve as core driving forces in skeletal aging. In this review, we introduce the concept of the Skeletal Aging Triangle, aiming to elucidate the intricate interactions among these three factors and their synergistic effects in the skeletal aging process. We provide a comprehensive overview of the mechanisms that trigger cellular senescence within bone tissue, the molecular pathways by which estrogen maintains bone homeostasis, the remote regulation of bone metabolism by the gut microenvironment, and the interconnections among these processes. A deeper understanding of the Skeletal Aging Triangle offers new perspectives for the prevention and treatment of skeletal aging–related diseases, while also contributing to improved patient quality of life and alleviating the healthcare burden on society.

## 1 Introduction

The global aging population significantly exacerbates the burden of osteoporosis, characterized by reduced bone mass and microstructural deterioration. Data shows that approximately nine million people worldwide are affected by osteoporotic fractures each year. It is further estimated that by 2050, the incidence of hip fractures in elderly men will increase by 310%, and by 240% in elderly women (Gullberg et al., 1997; Hurtado et al., 2024). This imposes substantial physical and economic challenges on patients and society.

While the maintenance of bone homeostasis was traditionally attributed primarily to estrogen regulation, recent studies reveal that bone aging results from complex multi-system interactions. Beyond estrogen deficiency alone, factors including cellular senescence, oxidative stress, and gut microbiome dysregulation form an intricate regulatory network. Unraveling these multi-factorial mechanisms is therefore essential for developing effective therapeutic strategies.

Based on current evidence, this review introduces the “Skeletal Aging Triangle,” establishing cellular senescence, estrogen deficiency, and gut microbiome dysregulation as the core drivers of bone aging. This framework highlights their synergistic interactions across spatial and regulatory levels. Specifically, cellular senescence serves as the local pathological basis, deteriorating the microenvironment via the senescence-associated secretory phenotype (SASP) (Kirichenko et al., 2025). Estrogen deficiency not only acts as the initiator of postmenopausal bone loss but also serves as the bridge connecting cellular status and systemic inflammation. Its decline accelerates bone marrow stromal cell senescence and weakens intestinal barrier function (Kverka and Stepan, 2024). Finally, the gut microbiome, as a central regulator of the body’s interface with the external environment, integrates metabolic signals through microbial metabolites (such as short-chain fatty acids), dynamically modulating the immune-inflammatory state of bone tissue and forming a “gut-endocrine-bone” axis for closed-loop regulation (Fu et al., 2024). This multi-dimensional, cross-scale interplay—spanning local cellular conditions, systemic endocrine imbalance, and remote gut regulation—forms the core framework driving bone aging. While oxidative stress, mitochondrial dysfunction, and chronic inflammation are critical, they act primarily as downstream effects converging within this network. Thus, the “Skeletal Aging Triangle” serves as both the center of synergistic interactions and the upstream hub integrating these mechanisms.

The innovative aspect of this review lies in the introduction and systematic argumentation of the “Skeletal Aging Triangle” as an integrative framework. Unlike previous studies that treat related factors independently, this work identifies cellular senescence, estrogen deficiency, and gut microbiome dysregulation as the three core driving forces. It emphasizes their synergistic interactions through the inflammation-immune axis, which elevates bone aging to the level of a systemic syndrome. Mechanistically, the review not only clarifies the independent pathways of each factor but also delves into their molecular interactions (such as estrogen resistance mediated by SASP) and underscores the spatiotemporal heterogeneity of their actions (for example, the initiation role of estrogen and the chronic core role of cellular senescence), providing a new perspective for understanding clinical heterogeneity.

The “Skeletal Aging Triangle” model is a significant advancement of existing multifactorial models. Its key improvement lies in: shifting from listing risk factors to distilling and correlating the three core driving forces; transitioning from a “parallel independence” understanding to revealing their dynamic interactions and malignant feedback loops; and introducing a dynamic weighting perspective to explain the phase-dependent and individual differences in the aging process. This framework not only deepens the integrated understanding of bone aging mechanisms but also lays the theoretical foundation for developing multi-targeted, synergistic intervention strategies.

Accordingly, this review will systematically discuss the independent mechanisms and complex interactions of each factor in the “Skeletal Aging Triangle,” and explore innovative treatment strategies and future directions for bone aging from this perspective.

## 2 Cellular senescence: the “time bomb” within bone tissue

### 2.1 Definition and core characteristics of cellular senescence

In 1961, Hayflick discovered that cultured human fibroblasts eventually cease dividing even under optimal conditions. This irreversible proliferative arrest, termed the “Hayflick limit,” represents a hallmark of cellular senescence (Hayflick and Moorhead, 1961).

Cellular senescence is an irreversible growth arrest triggered by replication exhaustion or stressors like DNA damage. Characterized by cell cycle arrest, it involves elevated regulators Cdkn2a (p16) and Cdkn1a (p21). Accordingly, targeting p16INK4a-positive cells has been proposed to delay aging-related pathologies, including osteoblast senescence. However, senescence markers exhibit context-dependent specificity. In the skeletal system, p16INK4a and p21 demonstrate distinct roles in chronic aging and acute injury repair. p16INK4a, a key driver of bone degeneration, significantly alleviates osteoporotic phenotypes when genetically cleared. In contrast, p21 shows limited specificity for age-related bone aging; long-term clearance of p21-positive cells in aged mice did not improve bone mass or reduce progenitor accumulation. Therefore, p16INK4a is a more reliable marker of age-related skeletal aging, while p21 plays a greater role in acute injury repair (Saul et al., 2024).

Additionally, while senescence-associated β-galactosidase (SA-β-Gal) is a standard marker, its sensitivity is limited by dependence on the GLB1 gene, potentially leading to false negatives. In contrast, senescence-associated α-L-fucosidase (SA-α-Fuc) has emerged as a more robust, GLB1-independent biomarker, particularly in mesenchymal stem cells (MSCs). Novel α-Fuc-responsive probes enable specific identification of senescent MSCs that evade SA-β-Gal detection. Thus, SA-α-Fuc serves as a reliable supplementary marker, although its specificity in terminally differentiated osteocytes requires further validation (Weng et al., 2022).

Another key feature of senescent cells is the SASP, which represents a hallmark of cellular senescence. SASP exerts both local paracrine and systemic effects, influencing the pathological and physiological functions of many senescent cells (Wang et al., 2024). SASP comprises a complex mixture of factors, including growth factors, chemokines, pro-inflammatory cytokines, and extracellular matrix (ECM) remodeling enzymes. Key SASP-associated proteins include tumor necrosis factor-α (TNF-α), monocyte chemoattractant protein (MCP)-1 and MCP-2, interleukins such as IL-6, IL-1α, IL-7, and IL-8, plasminogen activator inhibitor, growth-regulated oncogenes (GRO-α, GRO-β, GRO-γ), IGF-7, macrophage inflammatory protein-1α, and matrix metalloproteinases (MMPs), including MMP-1, MMP-10, and MMP-3 (Freund et al., 2010; Greene and Loeser, 2015). Additional SASP components include exosomes, vesicles, specific DNA fragments, diverse microRNAs, and other non-coding RNAs (Fang et al., 2023). Collectively, these factors contribute to the complexity and dynamic nature of SASP, influencing intercellular communication and regulating processes such as tumor microenvironment modulation, tissue repair, and aging. In the bone microenvironment, SASP exhibits significant heterogeneity, with IL-6, TNF-α, IL-1β, MCP-1, and MMP-13 identified as core components linking senescence to dysfunction. Quantitative analyses reveal that in 24-month-old mice, primary bone cells show elevated expression of IL-6 (∼2.5-fold), the matrix-degrading enzyme Mmp13 (∼4-fold), and the pro-aging factor Pai1 (∼5-fold) compared to young controls. Similarly, human iliac biopsies from elderly individuals exhibit ∼3-fold higher p16INK4a levels accompanied by increased pro-inflammatory cytokines (Farr et al., 2016). These factors drive excessive osteoclast activation via paracrine signaling and induce hematopoietic myeloid skewing through IL-1α and G-CSF, further exacerbating bone resorption (Li et al., 2023).

However, interventions targeting senescent cells face risks due to the “double-edged sword” role of senescence in homeostasis. First, transient senescence is critical for acute repair: senescent cells peak on day 4 post-injury, secreting PDGF-AA to induce myofibroblast differentiation. Premature clearance via senolytics (e.g., Dasatinib + Quercetin) delays healing and compromises bone callus strength by disrupting MSC recruitment. Second, senescence serves as a cancer suppression barrier; systemic inactivation of p53/p21 or p16INK4a pathways alleviates senescence phenotypes but significantly increases spontaneous tumor formation due to loss of cell cycle arrest (Li et al., 2023). Furthermore, while clearing p16INK4a-positive cells increases bone mass, indiscriminate targeting damages bone marrow vascular integrity, as these cells are essential for endothelial barrier function (Farr et al., 2016). Thus, distinguishing “pathogenic chronic” from “physiological acute” senescence is vital for clinical safety.

### 2.2 Triggers and hierarchical effects of osteocyte senescence

In bone tissue, various cell types (osteocytes, osteoblasts, osteoclasts, and bone marrow mesenchymal stem cells) are susceptible to senescence induced by intrinsic and extrinsic stressors. Traditionally, oxidative stress, telomere attrition, chronic mechanical overload, and pro-inflammatory signals are considered the main triggers of bone aging. Recent *in vivo* studies reveal these factors have hierarchical effects on skeletal aging:

Firstly, telomere attrition and the persistent DNA damage response (DDR) are regarded as the core drivers of *in vivo* bone aging. Experimental evidence confirms that in aged mice and human bone tissue, senescent osteocytes exhibit a noticeable accumulation of telomere-associated DNA damage foci (TIFs), marked by a significant increase in γH2AX foci co-localizing with telomeres. This telomere “uncapping” triggers persistent DDR signals, activating the ATM (Ataxia-telangiectasia mutated) and ATR kinase cascade, which subsequently transmits downstream signals to the p53/p21 and p16INK4a/Rb pathways, thereby inducing permanent cell cycle arrest (Farr et al., 2016; Li et al., 2023). Unlike the temporary DDR induced by acute injury, DDR resulting from telomere dysfunction is difficult to repair *in vivo*, and its persistence is critical for maintaining SASP expression (Lin and Epel, 2022; Gong et al., 2025). This sustained signaling not only leads to a marked increase in the secretion of RANKL and MMP13 by osteocytes, directly driving bone matrix degradation and osteoclast activation, but also interferes with the normal function of adjacent osteoprogenitor cells through paracrine effects (Farr et al., 2016; Li et al., 2023).

Secondly, immune signaling pathways, such as IL-1β, TNF-α, and NF-κB, act as key regulators and amplifiers, spreading the senescent phenotype through paracrine “bystander effects” in the bone marrow microenvironment (Li et al., 2023). This inflammatory environment not only maintains the continuous secretion of SASP through the cGAS-STING pathway by sensing DNA fragments in the cytoplasm (Li and Chen, 2018), but also induces a shift in the hematopoietic niche towards a myeloid bias (Mitchell et al., 2023), thereby increasing the osteoclast precursor reserve and further exacerbating the chronic malignant inflammatory cycle (Li et al., 2023). It is noteworthy that SASP’s regulatory role in osteoclastogenesis does not always exert a simple promoting effect; its function shows marked context-dependence and spatial specificity. In pathological stress models, SASP exhibits strong osteoclast-promoting activity: studies have shown that breast cancer cells can induce “premature senescence” in osteocytes, and these senescent osteocytes secrete large amounts of fibroblast growth factors and osteoclast-promoting factors (such as RANKL, MMP13), directly triggering bone destruction during the bone metastasis process (Kaur et al., 2024). However, in the context of natural aging, the role of localized SASP remains controversial. Research by Farr et al. revealed that while systemic clearance of senescent cells can effectively reduce osteoclast numbers in aged mice, specific local clearance of senescent osteocytes (local senolysis) only improves bone formation and microstructure but does not replicate the effect of reduced osteoclasts (OCs) observed with systemic clearance (Farr et al., 2023). This finding suggests that in natural aging, the localized SASP may not be the direct driver of osteoclast activation, and the enhanced osteoclastogenesis may depend more on systemic circulating SASP or senescent cells from other tissues. Therefore, the contribution of SASP to osteoclastogenesis depends on the aging-inducing factors (pathological stress vs. natural attrition) and the distribution of senescent cells.

Furthermore, oxidative stress induces excessive reactive oxygen species (ROS) production, which leads to DNA oxidative damage, including 8-OHdG, and participates in the regulation of cellular stress and damage response pathways (Vitale et al., 2023). If this oxidative DNA damage is not promptly repaired, it can become a permanent genetic damage signal. Simultaneously, oxidative stress also induces lipid peroxidation, further exacerbating cellular metabolic dysfunction by disrupting mitochondrial respiratory chains (Sadasivam et al., 2022).

Finally, the impact of mechanical loading shows significant localization and context-dependence. Osteocytes, as the primary mechanosensors, experience an imbalance in mechanotransduction (e.g., long-term mechanical unloading), which results in the loss of mitochondrial membrane potential and promotes osteocytes to secrete higher levels of Sclerostin and RANKL. This abnormal shift in biomechanical signaling not only directly mediates bone resorption but also accelerates the local microenvironment’s aging cascade (Verbruggen et al., 2023; Jin and Nolte, 2025).

This hierarchical effect at the molecular level is manifested through the close interaction and temporal sequencing between the p53/p21, p16INK4a/Rb, and NF-κB pathways. When telomere attrition or DNA damage activates ATM/ATR kinases, signals first pass through the p53/p21 pathway, inducing a temporary cell cycle arrest (Li et al., 2023). Subsequently, the expression of p16INK4a increases progressively with accumulated damage, acting as a “molecular lock” by inhibiting CDK4/6 and maintaining low phosphorylation of Rb proteins, thus locking the cell in permanent cycle arrest (Farr et al., 2016; Li et al., 2023). Meanwhile, NF-κB, the core transcription factor of SASP, is activated not only by upstream DDR signals (e.g., p38 MAPK pathways) but is also further reinforced by the cGAS-STING pathway sensing cytoplasmic DNA damage (Li et al., 2023; Kirichenko et al., 2025). It is important to note that these signaling pathways exhibit positive feedback loops: the pro-inflammatory cytokines (such as IL-6 and TNF-α) secreted by NF-κB can self-amplify by activating the transcription of p16INK4a and p21, thereby forming a self-sustaining senescence amplification effect in the bone microenvironment, ultimately resulting in an imbalance between RANKL/OPG and driving bone resorption (Farr et al., 2016; Josephson et al., 2019; Li et al., 2023).

### 2.3 Heterogeneity of senescence in major osteocyte subtypes

During bone aging, different cell populations within the bone microenvironment exhibit highly specific senescence-triggering mechanisms and phenotypic features. Bone marrow mesenchymal stem cells (BMSCs) are primarily triggered by chronic inflammation in the bone marrow niche (e.g., elevated IL-1β) and oxidative stress, and their senescent phenotype is marked not only by a shift in lineage differentiation towards myeloid and adipogenic directions but also by lysosomal dysfunction and disrupted lipid metabolism (Li et al., 2023; Kirichenko et al., 2025). As long-lived cells, osteocytes are mainly driven by accumulated telomere-related DNA damage foci (TIFs), and they serve as the core source of SASP in bone tissue. Experimental data show that in aged mice, mRNA levels of p16INK4a (approximately 2.5 times higher) and p21 (approximately 4 times higher) in primary osteocytes are significantly upregulated compared to younger controls. This leads to the secretion of high levels of RANKL, which enhances osteoclast activity and consequently results in excessive bone matrix degradation. In contrast, osteoblast senescence also involves an increase in p16INK4a levels but is primarily characterized by a downregulation of genes such as Runx2 and Osterix, which leads to impaired mineralization function, and the intensity of SASP secretion is quantitatively much weaker than in osteocytes (Farr et al., 2016). Additionally, OCs display a “senescence-like” hyperactive phenotype driven by the surrounding senescent microenvironment, characterized by prolonged lifespan and abnormal bone resorption activity due to continuous RANKL signaling, ultimately disrupting bone remodeling balance (Farr et al., 2016; Kirichenko et al., 2025).

### 2.4 Mechanisms by which cellular senescence drives skeletal aging

During aging, osteogenic lineages undergo profound changes that impair their ability to form, maintain, and repair bone. Progenitors of osteoblasts (OBs), including mesenchymal stem cells, exhibit reduced proliferative capacity and multipotency, leading to diminished osteogenic differentiation potential. In addition, MSC senescence plays a decisive role in bone mass maintenance. Senescent MSCs gradually lose their self-renewal ability and preferentially differentiate into adipocytes rather than OBs, leading to bone loss and fat accumulation. This shift toward adipogenic differentiation (Adipogenic shift) in MSCs has been widely validated *in vivo* and is considered a core pathological basis of age-related osteoporosis and “yellow marrowization.” Lineage tracing and single-cell RNA sequencing (scRNA-seq) studies have found significant reorganization of MSC subpopulations in the aging bone microenvironment, with a marked expansion of adipogenic progenitors (such as Malps) as age increases (Li et al., 2025). At the molecular level, this process is regulated by an imbalance between the Runx2 and PPARγ axes, where age-related intrinsic changes force MSCs to preferentially differentiate toward the adipogenic lineage (Weng et al., 2022). Moreover, interventional studies provide stronger causal evidence: selective clearance of senescent cells through Senolytics not only significantly reduces bone marrow fat accumulation but also effectively restores MSC osteogenic potential (Zhang et al., 2025). These multidimensional *in vivo* findings collectively establish the key role of MSC senescence in driving the bone marrow adipogenic shift.

Meanwhile, osteoclast precursors are increasingly recruited and differentiated under the influence of SASP factors secreted by senescent OBs, thereby enhancing osteoclast activity and bone resorption. For instance, IL-6 within SASP activates the JAK/STAT3 signaling pathway, promoting the differentiation of osteoclast precursors into mature OCs (Zhu et al., 2025), Notably, IL-6 in the bone microenvironment primarily drives osteoclastogenesis indirectly: it acts on OBs, BMSCs, or osteocytes through the IL-6 receptor (IL-6R), inducing these cells to secrete RANKL, which then promotes osteoclast differentiation, rather than acting directly on osteoclast precursors. Studies show that in osteoblast-specific IL-6R knockout mice, even in the presence of IL-6, the ability to promote osteoclast maturation is significantly reduced. Conversely, OBs overexpressing IL-6R enhance IL-6’s osteoclast-promoting effects. Additionally, the complex formed by IL-6 and its soluble receptor (sIL-6R) can promote JAK2 phosphorylation and induce osteoblast-like MLO-Y4 cells to secrete RANKL, which stimulates RAW264.7 cells to differentiate into OCs in co-culture systems (Zhu et al., 2025). Furthermore, TNF-α upregulates the expression of NF-κB receptor activator ligand, further driving osteoclastogenesis and bone resorption (Kitaura et al., 2022).

Aging osteocytes also disrupt the integrity of the osteocyte network through the secretion of SASP factors, thus promoting skeletal aging. This impairs the osteocytes’ ability to sense and transmit mechanical stress signals, disrupting bone remodeling. Mechanistically, SASP secreted by aging osteocytes can downregulate the expression of the key osteogenic transcription factor RUNX2 in BMSCs through paracrine signaling, directly inhibiting new bone formation. At the same time, the aging bone microenvironment (e.g., bone marrow adipose tissue) generates signals that promote osteoclastogenesis (such as RANKL), further disrupting the balance of bone remodeling and exacerbating bone loss (Zhang et al., 2025).

### 2.5 The role of cellular senescence and its multidimensional interactions

In the “Skeletal Aging Triangle,” cellular senescence represents the fundamental pathological basis and the primary driver of aging (“the oldest old”). While estrogen deficiency triggers rapid bone loss during the early postmenopausal transition, the accumulation of senescent cells (SnCs) and their associated SASP maintain a chronic “inflammatory” state, which, with advancing age, becomes a major determinant of bone health, surpassing the acute effects of hormonal withdrawal (Fuggle et al., 2025). Quantitative studies, based on age groups, have shown that in aged subjects (such as 24-month-old mice), the expression of senescence markers like p16INK4a and p21 in the bone microenvironment is significantly higher than in young controls (such as 3-month-old mice). The age-dependent accumulation of these SnCs is directly correlated with decreased mineral apposition rate (MAR) and bone formation rate (BFR), providing a quantifiable link between the degree of senescence and the severity of the osteoporotic phenotype (Zhang et al., 2025).

SASP factors secreted by senescent cells can interfere with estrogen signaling pathways, exacerbating the harmful effects of estrogen deficiency on bone metabolism. Cellular senescence antagonizes estrogen’s protective effects by reducing the availability of estrogen receptors and impairing the fidelity of downstream signaling. At the molecular level, senescent cells (SnCs) exhibit a significant reduction in the expression of SIRT6, a necessary nuclear deacetylase for bone homeostasis. The deficiency of SIRT6 in osteoclast precursors leads to the hyperacetylation of estrogen receptor α (ERα) and subsequent proteasomal degradation, thereby impairing ERα-mediated FasL expression and osteoclast apoptosis, resulting in enhanced bone resorption and bone loss (Moon et al., 2019). The upstream driver of this critical pathway is SASP. First, estrogen deficiency induces BMSCs to activate the JAK2/STAT3 signaling axis, triggering an inflammatory cascade of SASP factors (Wu et al., 2020). These SASP factors (such as TNF-α, IL-6) and the ROS they induce collectively shape the chronic inflammatory and oxidative stress environment (Weitzmann and Pacifici, 2006). This SASP-driven chronic inflammation and oxidative stress environment has been shown to significantly inhibit the expression and activity of key anti-aging proteins, such as Sirtuin 6 (Sirt6) in osteocytes (Zhao et al., 2025a). Secondly, SASP components further antagonize ERα’s biological activity at the transcriptional level by activating stress pathways, such as NF-κB (Demaria, 2019). Moreover, the high metabolic demands of the senescence phenotype lead to NAD+ depletion; this “NAD+ scavenging” effect damages the activity of other sirtuins (SIRT1/SIRT3), essentially creating a cellular estrogen resistance state (Kim et al., 2021).

In the gut, the accumulation of senescent intestinal stem cells (ISCs) and their persistent SASP-driven inflammatory milieu disrupt gut function and systemic homeostasis. For example, mice exposed to iron radiation exhibit elevated ROS and sustained DNA damage in ISCs, accompanied by premature senescence and accumulation of SASP markers (Kumar et al., 2019). Senescence also diminishes ISC proliferation, self-renewal, and motility, aggravating local inflammation and impairing gut maintenance (Nalapareddy et al., 2022). Furthermore, senescent cells influence the composition and function of the microbiome. Such interactions play a crucial role in the development of aging-related diseases, including musculoskeletal disorders, and present potential therapeutic targets. The accumulation of various senescent cell types in the gut—such as epithelial and immune cells—and their associated SASP may alter microbial diversity and microbiota-derived metabolites (Jang et al., 2024). These findings highlight the critical role of cellular senescence in the intestinal epithelium and its broader impact on gastrointestinal function.

**FIGURE 1:**
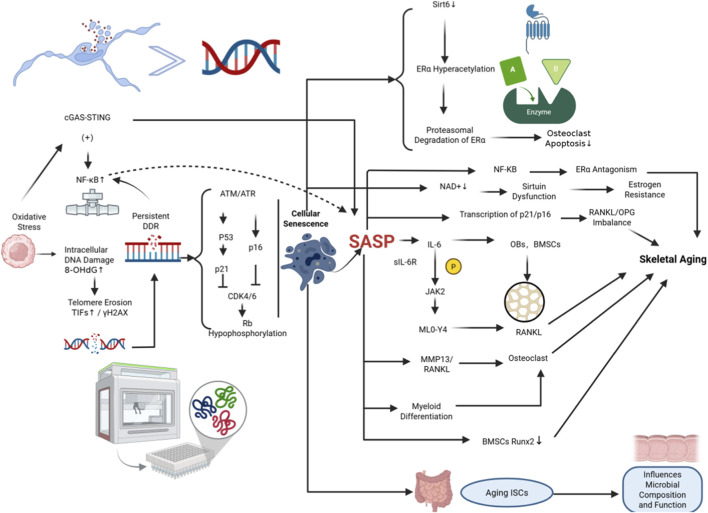
Mechanisms linking cellular senescence to skeletal aging. (Left) Oxidative stress and telomere dysfunction activate ATM/ATR and p53/p16INK4a pathways, triggering cellular senescence. (Right) The Senescence-Associated Secretory Phenotype (SASP) drives aging through three axes: 1) Bone Remodeling: SASP upregulates RANKL via JAK2 signaling while inhibiting Runx2, causing bone loss. 2) Estrogen Resistance: SASP depletes NAD+ and downregulates Sirt6, leading to ERα degradation and hormonal resistance. 3) Gut-Bone Axis: Senescence disrupts intestinal stem cells (ISCs) and microbial homeostasis, further exacerbating systemic aging.

Figure 1 integrates these mechanisms, illustrating how cellular senescence interacts with estrogen deficiency and the gut microbiome to drive skeletal aging.

## 3 Estrogen: the “guardian goddess” of bone and its decline

### 3.1 The central role of estrogen in maintaining bone homeostasis

Estrogen is indispensable for skeletal health, playing a central role in maintaining bone homeostasis. This homeostasis depends on a delicate balance between bone formation and resorption, mediated primarily by OBs and OCs. The equilibrium between OB- and OC-mediated bone metabolism is disrupted following the postmenopausal decline in estrogen levels.

OCs, which derive from the monocyte–macrophage lineage through mononuclear cell fusion, are critical for bone resorption. They degrade mineralized bone matrix and release minerals such as calcium and phosphorus into the bloodstream, thereby regulating skeletal remodeling. Estrogen exerts an inhibitory effect on OCs: it binds to estrogen receptors (ERs) on osteoclast precursors, reducing their generation, differentiation, and activity. Estrogen deficiency attenuates this suppression, resulting in increased numbers and activity of OCs, which accelerates bone resorption (Hsu et al., 2024).

OBs, in contrast, are bone-forming cells primarily derived from BMSCs (Nookaew et al., 2024). Estrogen promotes the differentiation of BMSCs into OBs, thereby increasing OB output. Under estrogen deficiency, fewer BMSCs undergo osteogenic differentiation, leading to reduced OB formation (Wang P. et al., 2023). In addition, binding of estrogen to ERs regulates OB activity, whereas estrogen deficiency diminishes OB proliferation and matrix synthesis.

Beyond stimulating OB differentiation, estrogen promotes OC apoptosis and suppresses osteoclastogenesis through multiple pathways. Estrogen enhances the production of osteoprotegerin (OPG) while inhibiting IL-1 and TNF, thereby reducing the release of macrophage colony-stimulating factor (M-CSF), receptor activator of NF-κB ligand (RANKL), and IL-6. In states of estrogen deficiency, bone resorption and formation become uncoupled: osteoclast-mediated resorption outpaces osteoblast-mediated formation, resulting in net bone loss. Additionally, estrogen deficiency triggers the hypersecretion of pro-inflammatory cytokines, which accelerates cellular senescence and promotes skeletal aging (Zhao et al., 2025a).

### 3.2 Mechanisms of estrogen-mediated bone protection

Estrogen exerts essential regulatory effects on cellular senescence and apoptosis. It mitigates oxidative stress (Sasaki et al., 2021), inhibits chondrocyte apoptosis, and modulates signaling pathways and proteins involved in cell survival. Estrogen regulates bone homeostasis through its classic nuclear receptors, ERα and ERβ, which display distinct spatial distribution in bone tissue. In human bone, ERα predominates in cortical bone, while ERβ is more prevalent in trabecular bone. This differential distribution likely reflects their distinct physiological roles in different bone microenvironments. Although both receptors are expressed, considerable evidence suggests that ERα is the key mediator of estrogen’s protective effects on bone cells, including OBs, OCs, and osteocytes. Gene knockout studies confirm that estrogen cannot prevent bone loss in mice lacking both ERα and ERβ, even with functional androgen receptors, highlighting the indispensable role of ERα signaling in maintaining bone homeostasis. Therefore, when investigating the effects of estrogen deficiency, particular attention should be given to the pathological consequences of the imbalance in ERα-driven signaling networks in cortical and trabecular bone (Weitzmann and Pacifici, 2006). Through ER activation, estrogen promotes chondrocyte proliferation, thereby delaying senescence and apoptosis. ERα is particularly critical as a regulator of these processes. By activating ERα and the G protein–coupled estrogen receptor (GPER), estrogen synergistically enhances chondrocyte proliferation and regeneration, reduces oxidative stress, and suppresses senescence and apoptosis. These protective effects are mediated, for example, through regulation of the Bcl-2/Bax ratio and modulation of PI3K/Akt/mTOR and MAPK pathways (Börjesson et al., 2013; Zhao et al., 2025a).

At the same time, estrogen maintains skeletal integrity by suppressing osteoclast formation and activation via multiple molecular mechanisms. Activated ERs directly interact with transcription factor NF-κB, blocking its DNA binding and thereby reducing IL-6 production. Estrogen also attenuates c-Jun N-terminal kinase (JNK) activity, decreasing activator protein 1 (AP-1) generation, which in turn inhibits TNF-α expression and reduces osteoclast sensitivity to RANKL. Additionally, estrogen suppresses casein kinase 2 (CK2) activity, diminishing the phosphorylation of early growth response factor 1 (Egr-1). Dephosphorylated Egr-1 shows increased affinity for transcription factor Sp-1, forming an Egr-1/Sp-1 complex, which decreases the level of free nuclear Sp-1 and ultimately downregulates M-CSF transcription, a key regulator of osteoclast differentiation. Together, these mechanisms—blocking NF-κB/IL-6 signaling, inhibiting JNK/AP-1–mediated TNF-α expression and RANKL responsiveness, and disrupting the CK2–Egr-1–Sp-1 axis in M-CSF transcription—synergistically suppress osteoclastogenesis and bone resorption, thereby maintaining the dynamic balance between bone resorption and formation (Weitzmann and Pacifici, 2006).

### 3.3 Interactions between estrogen and other factors

In the “Skeletal Aging Triangle,” estrogen deficiency is a primary initiator and driving factor in the early stages of skeletal aging, particularly prominent in postmenopausal women (Li et al., 2016). While cellular senescence contributes to the chronic decline in bone function, the sharp drop in estrogen levels during perimenopause directly disrupts bone remodeling. This hormonal shift acts as a “catalyst,” accelerating the progression of the other two pillars of the triangle: it promotes premature osteoblast senescence (Josephson et al., 2019) and increases intestinal permeability, thereby amplifying systemic pro-inflammatory signals (Li et al., 2016). Layered data from experimental models highlight the profound effect of estrogen levels on bone microstructure. In ovariectomized (OVX) mice, estrogen deficiency leads to rapid trabecular bone loss, with a sharp decline in spine bone volume fraction (BV/TV) within weeks. Following long-term steroid deprivation, osteoclast surface area and number significantly increase (Li et al., 2016).

Estrogen closely interacts with cellular senescence. By inhibiting oxidative stress, inflammatory responses, and cell cycle dysregulation, estrogen delays the progression of cellular senescence. In states of estrogen deficiency, markers of senescence are elevated, alongside chronic inflammatory responses and oxidative stress levels (Foster and Kumar, 2025). Estrogen deficiency leads to an increase in ROS, creating an oxidative stress environment that is a critical factor in the pathogenesis of postmenopausal osteoporosis. Mechanistically, estrogen deficiency downregulates SIRT1. The reduced activity of SIRT1 stabilizes and activates p53, thereby inducing p21-mediated cell cycle arrest and senescence. Furthermore, estrogen deficiency impairs mitochondrial function by decreasing SIRT3 expression, leading to reduced SOD2 deacetylation and further accumulation of mitochondrial ROS, which exacerbates the senescent phenotype (Zhao et al., 2025a). In ovariectomized (OVX) experimental models of estrogen deficiency, these molecular alterations are characterized by an increased proportion of SA-β-gal-positive cells and elevated expression of the senescence marker p16INK4a (Zhao et al., 2025a).

Estrogen also exerts significant influence on the gut microenvironment. Estrogen levels modulate intestinal motility, which indirectly shapes the gut microbiota (Cross et al., 2024). In turn, the gut microbiota impacts motility through metabolites and fermentation products (Waclawiková et al., 2022). Estrogen deficiency reduces the expression of tight junction proteins, compromising barrier integrity and leading to gut barrier dysfunction (Li et al., 2016; Shieh et al., 2020). Consequently, loss of sex hormones accelerates gut dysbiosis and intestinal barrier failure (Shieh et al., 2020; Salazar et al., 2023).

The reciprocal relationship between sex hormones and the gut microbiota has been demonstrated in multiple rodent studies, showing increased relative abundance of Firmicutes and Proteobacteria and decreased abundance of Bacteroidetes (Kverka and Stepan, 2024). In ovariectomized rats, microbial richness declined significantly as early as 4 weeks post-surgery, with pronounced shifts in β-diversity. These alterations were largely driven by significant increases in several bacterial taxa, including Ruminococcus gnavus, Lachnospiraceae 10-1, Lachnospiraceae A4, and *Lactobacillus* reuteri, and were characterized by strong inter-individual variability. Estrogen supplementation reversed these ovariectomy-induced dysbiotic changes, restoring microbial composition toward normal (Meng et al., 2021).

**FIGURE 2:**
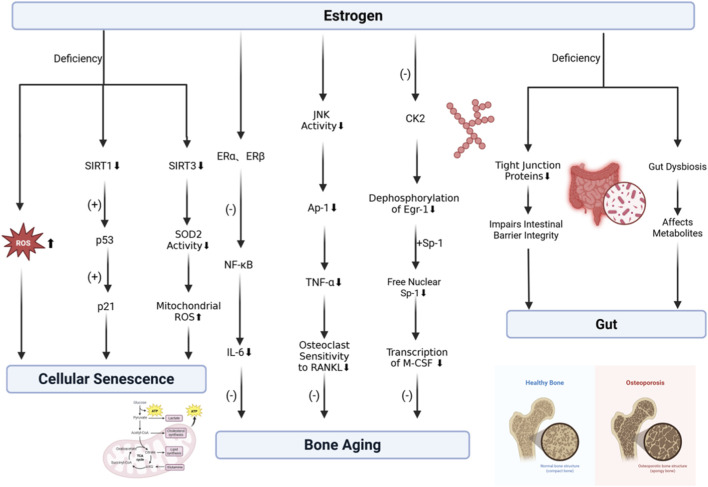
Molecular landscapes of estrogen-mediated skeletal protection. (Left) Estrogen deficiency drives cellular senescence via ROS accumulation and downregulation of the SIRT1/3-p53 signaling axis. (Middle) Estrogen exerts anti-resorptive effects through three mechanisms: 1) ERα/β-mediated NF-κB blockade; 2) JNK/AP-1 inhibition reducing TNF-α and RANKL sensitivity; 3) CK2/Egr-1/Sp-1 suppression lowering M-CSF transcription. (Right) Estrogen maintains gut homeostasis; deficiency compromises tight junction proteins and induces dysbiosis, exacerbating systemic aging.

Taken together, estrogen deficiency not only accelerates cellular senescence but also disrupts the gut microenvironment, impairing nutrient absorption and metabolism, and thereby indirectly compromising skeletal health. Figure 2 synthesizes these multidimensional interactions, illustrating how estrogen deficiency disrupts the delicate balance between cellular senescence, bone signaling pathways, and gut homeostasis.

## 4 Gut microenvironment: the “remote commander” of bone homeostasis

### 4.1 Core components and functions of the gut microenvironment

The gut microenvironment is a complex ecosystem primarily composed of the gut microbiota, intestinal mucosal barrier, intestinal immune system, and gut endocrine system. The human intestine harbors a highly diverse microbial community of more than 10^14^ microorganisms, encompassing over 2000 species—outnumbering human cells—and playing an indispensable role in maintaining overall health (Thursby and Juge, 2017). The gut microbiota contributes critically to digestion, nutrient absorption, immune function, and pathophysiological processes.

Intestinal epithelial cells (IECs), including intestinal stem cells (ISCs), goblet cells, and Paneth cells, are located at the interface between the luminal microbiota and host tissues (Hohman and Osborne, 2022). Beyond acting as a protective barrier, these epithelial cells regulate inflammatory balance within the gut.

As the body’s largest immune organ, the intestine is critically involved in autoimmune diseases. The gut microbiota, as a complex microbial ecosystem, is essential for host health and symbiotic interactions. While the host provides a habitat for microorganisms, the microbiota supplies nutrients and, importantly, elicits immune responses that protect against pathogens (Zegarra-Ruiz et al., 2021).

### 4.2 Evidence linking gut microenvironment dysregulation and bone

With aging, the gut microenvironment undergoes marked changes, including reduced microbial diversity, depletion of beneficial bacteria, enrichment of harmful taxa, impaired mucosal barrier function, and dysregulated intestinal immunity. Collectively, these alterations constitute gut dysbiosis. Such microbial imbalance leads to barrier dysfunction, increased intestinal permeability, and leakage of toxic bacterial metabolites into circulation, which promotes systemic low-grade inflammation and immune activation (Malesza et al., 2021). Accumulating evidence shows that these processes are closely associated with bone mass regulation, mechanical properties, hematopoiesis, development, metabolism, osteoporosis (Zheng et al., 2020), inflammation, fracture risk, and bone cancers.

The gut exerts multidimensional regulation of bone homeostasis through microbial metabolites, immune modulation, endocrine signaling, neural pathways, and even direct intercellular communication. Dysbiosis disrupts these processes, driving chronic inflammation and cellular senescence—two key pathological bases of skeletal aging (Fu et al., 2024).

Microbial metabolites play a central role as signaling molecules, forming a sophisticated gut-bone axis regulatory network. First, short-chain fatty acids (SCFAs) suppress inflammation by binding to G-protein–coupled receptors GPR41/GPR43 or inhibiting histone deacetylases (HDACs), thereby blocking NF-κB signaling (Smith et al., 2013). SCFAs also bidirectionally regulate bone remodeling: promoting regulatory T cell (Treg) differentiation to suppress osteoclastogenesis while simultaneously activating Wnt10b signaling to enhance osteoblastogenesis (Li et al., 2021). SCFAs enhance the solubility and uptake of minerals such as calcium and phosphorus by lowering intestinal pH, which increases mineral ionization. In addition, they improve epithelial permeability and upregulate specific transporters, thereby facilitating transcellular transport. SCFAs also contribute to maintaining intestinal morphology and function, expanding the absorptive surface area and further enhancing mineral bioavailability (Wang et al., 2022), while microbial-derived bioactive peptides such as casein phosphopeptides act as calcium carriers to improve utilization (Liu G. et al., 2021). The microbiota also participates in the conversion of thyroxine (T4) to its active form triiodothyronine (T3), which promotes osteoblast proliferation via fibroblast growth factor receptor 1 (FGFR1) (Kanatani et al., 2004).

Furthermore, bile acid metabolites, particularly secondary bile acids such as lithocholic acid (LCA) and ursodeoxycholic acid (UDCA), exert bone-protective effects by activating their receptor TGR5. TGR5 signaling inhibits NF-κB nuclear translocation in osteoclast precursors, thus blocking RANKL-induced osteoclast differentiation (Li et al., 2021; Guan et al., 2023). Additionally, tryptophan metabolites, particularly indoles and their derivatives, modulate immune function by activating the aryl hydrocarbon receptor (AhR), promoting Treg cell differentiation and inhibiting pro-inflammatory Th17 cell activity, thereby maintaining bone immune balance. However, when the intestinal barrier is compromised, microbial-associated molecular patterns (MAMPs) translocate and become key drivers of bone loss. Metabolites such as lipopolysaccharides (LPS) activate MyD88-dependent pathways in bone marrow macrophages via TLR4, inducing the release of pro-inflammatory cytokines such as IL-6 and TNF-α, which further amplify local oxidative stress and cellular senescence in bone tissue (Li et al., 2021; Chen et al., 2025).

In summary, gut dysbiosis disrupts metabolic signaling networks and barrier function, creating a pro-inflammatory systemic and local bone microenvironment. This environment directly polarizes macrophages to the M1 phenotype through pro-inflammatory cytokines such as IL-6 and TNF-α, inhibiting osteoblastogenesis and promoting osteoclastogenesis (Fu et al., 2024). Moreover, the Treg/Th17 balance is modulated by the microbiota: Tregs suppress osteoclast differentiation and enhance Wnt10b-mediated osteogenesis (Fu et al., 2024), while Th17 cells exacerbate bone resorption through RANKL signaling.

In the aging microenvironment, inflammation accelerates immune cell senescence, creating a vicious cycle of “inflammaging.” The microbiota also influences estrogen metabolism: estrogen deficiency directly promotes bone loss, whereas probiotic supplementation (e.g., *Lactobacillus* acidophilus) alleviates estrogen-deficiency–induced osteoporosis. In addition, bacterial extracellular vesicles (bEVs) carry bioactive molecules with direct skeletal effects. For instance, Akkermansia muciniphila–derived bEVs attenuate osteoporosis (Liu J.-H. et al., 2021), while *Lactobacillus* animalis bEVs protect against glucocorticoid-induced osteonecrosis (Chen et al., 2022).

Aging is characterized by reduced microbial diversity (e.g., altered Firmicutes/Bacteroidetes ratio), depletion of probiotics (e.g., Bifidobacterium), and enrichment of pathobionts (e.g., Proteobacteria) (Fu et al., 2024). These shifts reduce SCFA synthesis, disrupt bile acid metabolism, and promote LPS accumulation, all of which induce systemic chronic inflammation (Fu et al., 2024). Notably, the role of Proteobacteria in skeletal aging is far from straightforward, exhibiting significant heterogeneity and controversy with a complex “double-edged sword” effect. Traditionally, excessive expansion of Proteobacteria is considered a hallmark of gut dysbiosis and inflammaging. In age-related pathological states, certain Proteobacteria members (e.g., *Escherichia coli* carrying the pks toxin) can induce DNA damage, triggering pro-inflammatory responses, damaging the intestinal barrier, and exacerbating systemic bone resorption (Qu et al., 2023). However, recent cross-cohort studies on centenarians challenge this singular view. These studies show consistent enrichment of specific Proteobacteria (e.g., Desulfovibrio, especially D. fairfieldensis) in the gut of centenarians, with Mendelian randomization analyses suggesting a significant positive correlation with longevity traits. Functional data indicate that these specific Proteobacteria members may participate in remote regulation of bone metabolism through the biosynthesis of vitamin K2 (menaquinone), exerting a potential bone-protective effect (Chen et al., 2024). This “Proteobacteria paradox” highlights that the gut microbiome’s impact on skeletal aging depends not only on phylum-level abundance changes but also on the metabolic characteristics of specific strains and their bioactive compounds.

**FIGURE 3:**
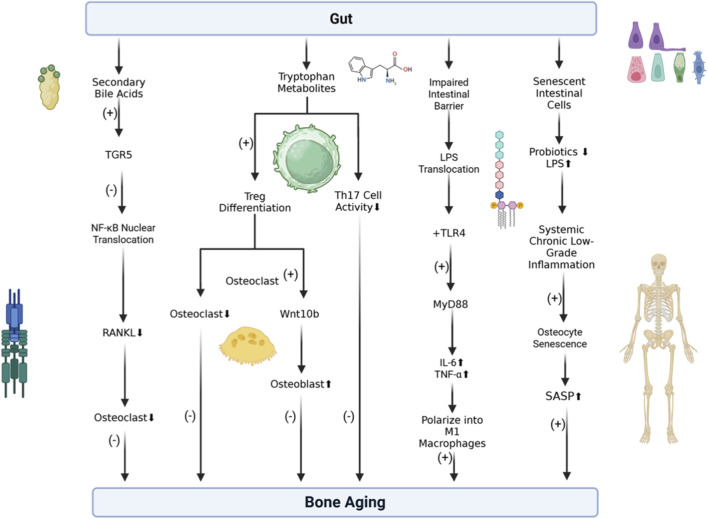
Microbial metabolites and immune signaling in bone homeostasis. (Left) Secondary bile acids activate TGR5 receptors to inhibit NF-κB and osteoclastogenesis. (Middle) Tryptophan metabolites activate AhR, promoting Treg differentiation while suppressing Th17 activity to balance bone remodeling. (Right) Dysbiosis increases intestinal permeability, allowing LPS translocation to activate the TLR4/MyD88 pathway, inducing M1 macrophage polarization and pro-inflammatory cytokine release that drives osteocyte senescence.

The chronic inflammatory environment induced by gut dysbiosis accelerates the senescence of OBs, macrophages, and bone stem cells. These cells release senescence-associated secretory phenotype factors (e.g., IL-6, GCA), further amplifying inflammation, inhibiting bone formation, and accelerating degenerative bone diseases such as osteoporosis and osteoarthritis. Specifically, specific microbial metabolites such as secondary bile acids and tryptophan derivatives, alongside pathogen-associated molecular patterns (PAMPs), orchestrate these immunomodulatory effects (Figure 3).

### 4.3 The role and multidimensional interactions of the gut microenvironment

In the “Skeletal Aging Triangle,” the gut microenvironment acts as a systemic amplifier and remote regulator of bone metabolism. While cellular senescence and estrogen deficiency are local or hormone-driven factors, gut dysbiosis serves as a “bridge” connecting environmental factors (such as diet and medication) to bone health. This role is particularly prominent in individuals with metabolic comorbidities or chronic inflammation. The gut microbiome functions as a system amplifier: dysbiosis enhances cellular senescence through the secretion of pro-inflammatory metabolites and exacerbates the effects of estrogen deficiency on bone by disrupting the enterohepatic circulation of detrimental hormones (Fu et al., 2024).

The gut microbiota includes the so-called “estrobolome,” which expresses β-glucuronidase enzymes capable of converting conjugated estrogen into its active form, thus promoting enterohepatic recycling and systemic availability of estrogen (Fu et al., 2024). Dysbiosis, particularly reduced microbial diversity, impairs this process, leading to decreased active estrogen levels. Additionally, gut bacteria can biotransform polycyclic aromatic hydrocarbons and other pollutants into estrogen-like compounds, disrupting endocrine homeostasis (Van de Wiele et al., 2005).

Studies indicate that individuals with hormone deficiency often exhibit decreased bone mineral density accompanied by enrichment of Ruminococcus, whereas long-lived populations show increased abundance of Akkermansia and Bifidobacterium, which may help maintain metabolic homeostasis and delay aging. In contrast, *Fusobacterium* nucleatum has been linked to negative health outcomes (Kverka and Stepan, 2024).

Gut dysbiosis also disrupts tight junction proteins, increasing intestinal permeability and allowing LPS and microbial metabolites to enter circulation. This triggers systemic low-grade inflammation, which accelerates cellular senescence (Jang et al., 2024). The inflammatory microenvironment induces the release of SASP factors (e.g., IL-6, TNF-α), promoting senescence of bone and immune cells (Fu et al., 2024). Aging further exacerbates this by depleting anti-inflammatory bacteria (e.g., Bifidobacterium, butyrate-producing Clostridia) while enriching pro-inflammatory taxa (e.g., Proteobacteria), thereby amplifying inflammation and oxidative stress and driving cellular senescence (Jang et al., 2024).

Specific studies stratified by health status show significant differences in the gut microbiome profile between osteoporosis patients and healthy controls (Huang et al., 2022). Clinical evidence from an aged rat model (e.g., 20-month-old) indicates that the Firmicutes/Bacteroidetes (F/B) ratio, a marker of systemic dysbiosis, is significantly higher compared to younger rats (3 months old). This shift is directly associated with increased intestinal permeability and elevated serum levels of bone resorption markers, such as RANKL (Kverka and Stepan, 2024).

**FIGURE 4:**
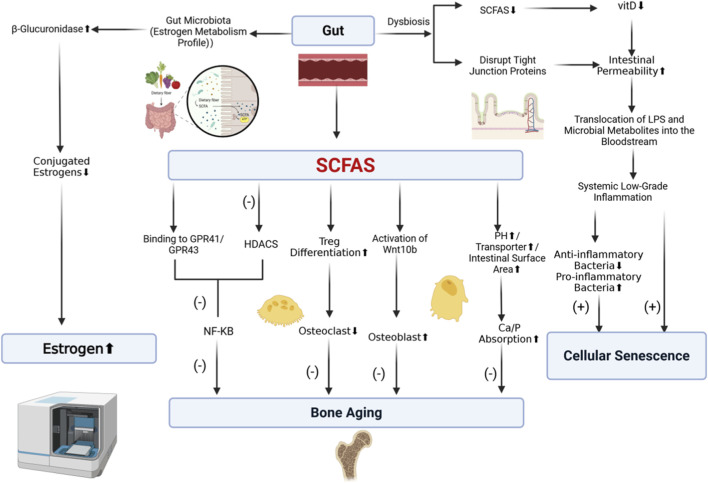
The integrative Gut-Bone axis: Estrobolome, SCFAs, and Senescence. (Left) The “Estrobolome” (via β-glucuronidase) reactivates conjugated estrogens, maintaining systemic hormone levels. (Middle) SCFAs exert pleiotropic protective effects by inhibiting HDACs, activating Wnt10b, and enhancing mineral absorption. (Right) Dysbiosis disrupts these protective pathways and compromises the intestinal barrier, triggering systemic inflammation that accelerates cellular senescence.

Gut microbiota-derived metabolites, such as SCFAs, upregulate the vitamin D receptor (VDR), a process crucial for maintaining intestinal barrier integrity and suppressing inflammation. Conversely, dysregulation of this pathway—due to gut dysbiosis with reduced SCFA production or vitamin D deficiency—exacerbates intestinal permeability (“leaky gut”) and chronic inflammation. This establishes a “gut microbiota-vitamin D-inflammation” axis, which indirectly influences estrogen activity and accelerates cellular senescence, thereby contributing to the pathogenesis of osteoporosis (Kverka and Stepan, 2024). Finally, Figure 4 illustrates the integrative “Gut-Bone-Estrogen” axis, highlighting how the estrobolome and SCFAs cooperatively maintain skeletal integrity against aging.

## 5 Therapeutic strategies and future perspectives targeting the “skeletal aging triangle”

### 5.1 Senotherapy targeting cellular senescence

Therapeutic strategies targeting cellular senescence have become an important research direction for preventing skeletal aging. Among various approaches, senolytics, which selectively eliminate senescent cells, have shown significant therapeutic potential. Dasatinib combined with quercetin (D + Q) is the most widely used combination in preclinical and early clinical studies. Research shows that in a focal radiation-induced bone loss model, D + Q significantly reduces the burden of senescent cells in the bone marrow, evidenced by decreased TIF^+^ OBs and osteocytes, downregulation of senescence markers (p16INK4a and p21), and suppression of several senescence-associated secretory phenotype (SASP) factors, ultimately promoting bone structural recovery, including improvements in bone mass and trabecular number and thickness. Therefore, D + Q has been shown to effectively reduce senescent cell burden in the bone marrow, alleviating both natural aging and focal radiation-induced bone loss (Chandra et al., 2020).

Although D + Q is effective, concerns have been raised about its broad-spectrum clearance potentially lacking tissue specificity. In contrast, natural flavonoids like fisetin, with better safety and low cytotoxicity, show unique advantages in reducing senescence-related markers and providing long-term preventive intervention. The core mechanism of fisetin as a senolytic drug lies in selectively targeting anti-apoptotic proteins (SCAPs) such as Bcl-2 and Bcl-xL, which are highly expressed in senescent cells, inducing senolysis. Additionally, fisetin downregulates SASP through inhibition of NF-κB signaling, alleviates chronic inflammation, and may exert protective effects through antioxidant activity and regulation of SIRT1, PI3K/Akt/mTOR pathways (Tavenier et al., 2024). Furthermore, senomorphics like melatonin reverse premature senescence of BMSCs via SIRT1-dependent pathways (Zhou et al., 2015), offering another approach to maintaining bone homeostasis.

To achieve more precise intervention and overcome the common off-target toxicity, such as thrombocytopenia, associated with traditional small molecule inhibitors (e.g., BCL-XL inhibitor Navitoclax), emerging proteolysis-targeting chimeras (PROTACs) technology has been developed. By inducing ubiquitin-mediated degradation of target proteins instead of merely inhibiting their function, PROTACs not only maintain efficient clearance activity but also significantly improve the safety profile and tissue selectivity of the drugs. At the same time, chimeric antigen receptor T (CAR-T) cell therapy targeting the uPAR protein on senescent cells’ surface and β-galactosidase-responsive selective delivery systems offer diverse cutting-edge methods to precisely target senescent cells with high SA-β-Gal activity (He et al., 2023).

Another promising approach is to target senescence-associated antigens. Vaccines based on virus-like particles (VLPs), such as those directed against NGF, IL-1β, or TAU, can elicit immune responses that clear senescent cells and relieve osteoarthritis-associated pain. Moreover, inhibition of the senescence-associated secretory phenotype (SASP) has been explored, for instance by using TNF-α inhibitors or IL-1 receptor antagonists to block the release of inflammatory cytokines, thereby mitigating bone loss (Li et al., 2025).

Mesenchymal stem cell (MSC) transplantation has shown multi-mechanistic therapeutic potential in treating bone aging diseases such as osteoporosis. MSCs not only directly differentiate into OBs and chondrocytes to replenish functional cells but also secrete various growth factors such as BMP-2, IGF-1, and VEGF through paracrine signaling, promoting local repair and angiogenesis. At the same time, MSCs possess significant immunomodulatory capabilities, inhibiting pro-inflammatory factor secretion and regulating macrophage polarization, thereby reducing chronic inflammation’s damage to the bone microenvironment. Additionally, transplanted MSCs improve the age-related stem cell microenvironment by regulating signaling pathways such as Wnt/β-catenin and PPAR-γ, inhibiting bone marrow adipogenesis, maintaining extracellular matrix homeostasis, and delaying or even reversing local cell aging processes through mechanisms like activating sirtuin family proteins, enhancing antioxidant capacity, and promoting mitochondrial autophagy, thus facilitating tissue regeneration and functional recovery at multiple levels (Weng et al., 2022). Umbilical cord–derived extracellular vesicles (UC-EVs) carrying proliferating cell nuclear antigen (PCNA) can extend telomeres and promote the regeneration of senescent BMSCs (Lei et al., 2021).

Gene regulation strategies represent another important approach for intervening in skeletal aging, with miRNA-based regulatory networks being particularly crucial. During aging, pro-aging miRNAs (such as miR-146a, miR-183-5p, miR-34a) are upregulated. These miRNAs accelerate BMSC aging and osteogenic differentiation impairment by inhibiting stem cell proliferation, promoting cell cycle arrest, and enhancing SASP, leading to bone loss. In contrast, anti-aging miRNAs (such as miR-19a-3p, miR-20a) are downregulated during aging. These miRNAs help maintain stem cell vitality, suppress the expression of aging markers like p16INK4a/p21, and promote bone formation by activating osteogenic signaling pathways such as BMP2/SMAD. Therefore, inhibiting pro-aging miRNAs or supplementing anti-aging miRNAs using oligonucleotide drugs or delivery systems can reshape the gene expression profile in aging bones, improve stem cell function, and delay or reverse the aging process of the skeletal system. This provides potential molecular targets for developing novel therapies for age-related bone diseases such as osteoporosis and delayed fracture healing (He et al., 2023).

### 5.2 Estrogen-related therapies

Estrogen replacement therapy (HRT) directly restores estrogen levels, reverses gut dysbiosis, repairs intestinal barrier integrity, and inhibits bone resorption. Clinical studies have confirmed that HRT reduces the risk of postmenopausal osteoporosis, decreases the secretion of SASP factors from senescent cells (Zhao et al., 2025a), and enhances calcium absorption by upregulating the vitamin D receptor (VDR). Estrogen also maintains the expression of tight junction proteins by activating intestinal estrogen receptors (ERα/ERβ, GPER), thereby reducing bacterial translocation and systemic inflammation (Kverka and Stepan, 2024). Although estrogen is effective in maintaining bone homeostasis, its clinical application is limited by potential systemic risks. Studies have shown that the risks of estrogen therapy are formulation-dependent: for women with an intact uterus, estrogen alone increases the risk of endometrial hyperplasia and cancer, requiring the addition of progestogens to protect the endometrium. However, combined estrogen-progestogen therapy (EPT) may elevate the risk of breast cancer, while estrogen-only therapy has not shown the same level of association in some studies. Additionally, systemic estrogen application is associated with an increased risk of venous thromboembolism (VTE) and stroke, which is linked to its promotion of hepatic clotting factor synthesis. Therefore, clinical practice emphasizes personalized treatment, including the use of the lowest effective dose, optimizing the route of administration (such as transdermal delivery), and carefully assessing medical history to balance bone protection benefits with systemic risks. To overcome these challenges, the next-generation of selective estrogen receptor modulators (SERMs) has emerged, aiming to selectively target and activate ERα or ERβ, with the goal of preserving bone protection while minimizing adverse effects on tissues such as the breast and endometrium. Although most of these drugs are still in preclinical stages, they represent an important direction for developing safer and more precise therapies in the future (Wang L.-T. et al., 2023).

In addition to pharmacological interventions, non-pharmacological therapies have shown benefits. Aerobic exercise (e.g., walking or cycling) elevates central β-endorphin and estrogen levels, thereby reducing inflammatory cytokines such as TNF-α and IL-6. Resistance training increases estradiol levels, decreases C-reactive protein and inflammatory mediators, and alleviates pain. Mind-body practices (e.g., Tai Chi, Qigong, Baduanjin) help regulate estrogen levels while suppressing pro-inflammatory cytokines. Physical factor therapies, such as low-intensity pulsed ultrasound, stimulate ovarian secretion of growth differentiation factor 9 (GDF9) and bone morphogenetic protein 15 (BMP15), while inhibiting IL-1β and TNF-α. Transcutaneous electrical nerve stimulation at acupoints such as Guanyuan and Sanyinjiao enhances estrogen and endorphin secretion, while lowering cortisol and inflammatory factors. Traditional Chinese medicine approaches have also been investigated: acupuncture promotes ovarian estrogen secretion (E2) via activation of the hypothalamic-pituitary-ovarian axis, whereas moxibustion activates the PI3K/AKT pathway to enhance estrogen receptor expression and serum E2 levels. Finally, dietary interventions offer a complementary strategy. Phytoestrogens (e.g., soy products, flaxseed, chickpeas) bind to estrogen receptors, bidirectionally modulate estrogen levels, and inhibit osteoclast activity. Vitamin D regulates aromatase activity to promote estrogen synthesis, while trace minerals such as copper and zinc participate in the enzymatic metabolism of estrogen (Zhao et al., 2025b).

### 5.3 Modulating the gut microenvironment

Therapeutic strategies for osteoporosis are currently shifting from simple microbiota supplementation to more targeted mechanistic regulation. In probiotic interventions, supplementing specific probiotics such as Prevotella histicola has been shown to enhance gut barrier integrity by significantly upregulating the expression of tight junction proteins (e.g., ZO-1 and Occludin), thereby preventing the leakage of pro-inflammatory cytokines (TNF-α and IL-1β) into the circulation. This barrier repair effect further reduces the RANKL/OPG ratio in bone tissue, effectively inhibiting osteoclast activity and preventing estrogen deficiency-induced bone loss (Wang et al., 2021). Additionally, metabolites secreted by probiotics (such as *Lactobacillus* fermentum) show anti-aging potential by downregulating cell cycle inhibitors (e.g., p53-p21 and p16INK4a) in adipocyte precursors, and inhibiting the production of senescence-associated secretory phenotype (SASP) factors through blocking the NF-κB transcriptional pathway. Fecal microbiota transplantation (FMT) from young healthy donors not only restores gut homeostasis but also reverses age-related systemic inflammation (e.g., lowering serum TNF-α and IL-6 levels), improving bone health through the gut-bone axis (Jang et al., 2024).

Microbial metabolites, as key signaling molecules linking the gut to the skeleton, have shown great therapeutic potential. SCFAs such as butyrate, in addition to reducing inflammation by inhibiting the NF-κB pathway in intestinal epithelial cells, induce the secretion of glucagon-like peptides-1 (GLP-1) and GLP-2 from enteroendocrine L-cells, both of which have been shown to promote bone formation. SCFAs also stimulate the production of insulin-like growth factor 1 (IGF-1), directly supporting bone growth and remodeling (Kverka and Stepan, 2024). In bile acid metabolism, dual-targeting bile acid membrane receptor TGR5 and nuclear receptor FXR has demonstrated excellent anti-osteoporotic efficacy. Novel dual agonists, such as SH-479, inhibit osteoclast differentiation by activating the TGR5-cAMP-AMPK signaling axis, and block NF-κB phosphorylation and nuclear translocation. At the same time, activation of FXR promotes osteoblastogenesis, achieving bidirectional regulation of bone metabolism (Li et al., 2019). Secondary bile acids (e.g., isoalloLCA) exert anti-inflammatory and bone-protective effects by modulating the differentiation balance of Th17 and Treg cells in the gut’s lamina propria (Jang et al., 2024).

Phage therapy selectively eliminates specific pathogens associated with chronic inflammation or autoimmune diseases (such as certain pathogenic *Klebsiella* or *Enterococcus* species), thereby “modifying” the gut microbiome to reduce systemic inflammatory burden. Therapeutic phages are typically selected from lytic phages, which efficiently lyse host bacteria without producing lysogenic residues, making them suitable for clearing resistant strains or disrupting biofilms causing persistent infections. This precise strategy of eliminating pro-inflammatory bacteria through phage therapy provides a novel targeted intervention for preventing bone aging induced by gut microenvironment dysbiosis (Strathdee et al., 2023).

Moreover, estrogen replacement therapy plays a unique role in restoring the gut microenvironment by reducing bone resorption through blocking the abnormal migration of T cells from gut-associated lymphoid tissue to the bone marrow, a process typically driven by IL-17 and TNF-α. Supplementing with vitamin D enhances the gut barrier by upregulating antimicrobial peptide expression, maintaining the integrity of the mucus layer and tight junctions, and inhibiting Th17 cell differentiation, thus blocking inflammation-mediated bone loss (Kverka and Stepan, 2024).

### 5.4 Integrated intervention strategies: targeting the synergistic effects of the “triangle”

Given the complex interactions of the three factors in the “Skeletal Aging Triangle,” therapies targeting a single point often show limited efficacy. In contrast, comprehensive interventions based on the dimensions of the “Skeletal Aging Triangle” have shown significant advantages in enhancing efficacy and reducing clinical risks. These advantages are mainly reflected in three aspects:

First, at the mechanistic level, comprehensive strategies achieve synergistic blockage. Estrogen deficiency not only directly impairs bone metabolism but also induces gut barrier dysfunction and bacterial translocation, triggering systemic inflammaging, a pathway that cannot be fully blocked by single bone metabolism-regulating drugs (Kverka and Stepan, 2024). Clinical evidence shows that elevated gut permeability markers (such as FABP2) in perimenopausal women are directly associated with systemic inflammation and decreased bone density, confirming the core role of the “gut-bone axis” in human osteoporosis progression (Shieh et al., 2020). Furthermore, gut dysbiosis actively worsens metabolic dysfunction after hormonal fluctuations, rather than merely being a passive result of hormone decline, suggesting that estrogen replacement therapy (HRT) alone, without repairing the gut microenvironment, will be hindered by persistent metabolic disturbances (Cross et al., 2024).

Second, comprehensive interventions block the self-amplifying cycle of aging through multiple pathways. Cellular senescence and gut dysbiosis form a systemic link through SASP and microbial metabolites. Comprehensive interventions can simultaneously eliminate local “toxic sources” and restore systemic metabolic homeostasis (Jang et al., 2024).

Finally, from a risk control perspective, comprehensive strategies help achieve precise and safe intervention. By precisely regulating Treg cell-mediated immune responses through microbial metabolites (such as SCFAs), these strategies offer safer immune protection for the bone microenvironment, enhancing anti-osteoporotic effects while reducing dependence on high doses of single drugs and their potential side effects (Fu et al., 2024; Xu et al., 2025).

Thus, this multi-dimensional intervention strategy targets not only the local bone but also reshapes the “gut-endocrine-immune” network, transitioning from single-symptom improvement to systemic functional restoration (Fu et al., 2024).

However, the mechanisms remain unclear, requiring further animal and clinical studies to confirm efficacy, safety, and optimal timing.

## 6 Discussion and future perspectives

### 6.1 Summary of core conclusions

In summary, cellular senescence, estrogen deficiency, and gut microbiome dysbiosis together form an interactive network that collaboratively drives skeletal aging. Chronic low-grade inflammation and immune imbalance are the central hubs of this network: estrogen deficiency weakens the body’s anti-inflammatory barrier and accelerates bone cell senescence; the senescence-associated secretory phenotype (SASP) factors further elevate systemic inflammation; and the chronic inflammatory environment damages the intestinal barrier, leading to dysbiosis and the translocation of endotoxins (such as LPS). The latter not only directly stimulates osteoclast activity but also exacerbates systemic and local inflammation and oxidative stress via circulation, thus feeding back to accelerate further cellular senescence and inhibit estrogen signaling. In this way, the three factors catalyze each other through the inflammation-immune axis, forming a self-reinforcing vicious cycle. Therefore, effective interventions for skeletal aging must focus on breaking this cyclical network, rather than targeting a single node.

This “triangular” interaction framework provides a more integrated perspective for understanding the pathophysiology of postmenopausal osteoporosis and helps to clarify a long-standing controversy: does estrogen deficiency in postmenopausal bone loss involve estrogen-independent mechanisms? The evidence integrated in this review supports the affirmative answer. Although estrogen deficiency acts as a powerful “initiator” and “accelerator,” both cellular senescence and gut microbiome dysbiosis possess independent pathological pathways that do not rely on estrogen. For instance, telomere attrition-driven bone cell senescence or gut microbiome dysbiosis-induced systemic inflammation can independently lead to bone loss. Clinical observations, such as the limited efficacy of estrogen replacement therapy in some patients and the improvement of bone health by gut microbiome interventions without altering serum estrogen levels, support the existence of these parallel pathways. Therefore, the value of the “triangular” model lies in redefinition: postmenopausal osteoporosis should be seen not merely as a “single hormone deficiency disease,” but as a systemic syndrome driven by endocrine imbalance, cellular intrinsic aging, and remote ecological dysregulation, with multiple input nodes. This “triangular” interaction framework provides a more integrated perspective for understanding the pathophysiology of postmenopausal osteoporosis and helps to clarify a long-standing controversy: does estrogen deficiency in postmenopausal bone loss involve estrogen-independent mechanisms? The evidence integrated in this review supports the affirmative answer. Although estrogen deficiency acts as a powerful “initiator” and “accelerator,” both cellular senescence and gut microbiome dysbiosis possess independent pathological pathways that do not rely on estrogen. For instance, telomere attrition-driven bone cell senescence or gut microbiome dysbiosis-induced systemic inflammation can independently lead to bone loss. Clinical observations, such as the limited efficacy of estrogen replacement therapy in some patients and the improvement of bone health by gut microbiome interventions without altering serum estrogen levels, support the existence of these parallel pathways. Therefore, the value of the “triangular” model lies in redefinition: postmenopausal osteoporosis should be seen not merely as a “single hormone deficiency disease,” but as a systemic syndrome driven by endocrine imbalance, cellular intrinsic aging, and remote ecological dysregulation, with multiple input nodes.

This review systematically analyzes the mechanisms of each factor in the “triangle” and their interactions, and looks forward to integrated therapeutic strategies and future research directions. The aim is to provide a new theoretical framework and translational ideas for the prevention and treatment of skeletal aging and related diseases. The integrated intervention strategies targeting this “triangle,” whether single-agent or combination approaches, offer new opportunities and potential targets for the prevention and treatment of bone aging-related diseases.

### 6.2 Current limitations and challenges

Despite progress, current research on the “skeletal aging triangle” faces several key limitations. First, there are gaps in understanding the causal interactions, particularly regarding the gut microbiome’s regulation of estrogen levels. While the “estrobolome” hypothesis suggests gut bacteria may influence estrogen circulation, current evidence is mostly indirect, lacking direct *in vivo* causal data. Studies show gut microbiome modulation improves bone health but does not restore serum estrogen levels, indicating that its effects are likely mediated through immune-inflammatory pathways rather than by elevating estrogen (Guan et al., 2023; Guo et al., 2023; Hu et al., 2023). Thus, whether the microbiome is a “driver” or “responder” to estrogen in postmenopausal osteoporosis remains unclear.

Second, there is no standardized method to identify and quantify senescent cells in the skeletal system, limiting our understanding of their spatiotemporal dynamics and interaction with estrogen deficiency and gut dysbiosis. Although xINK4a and p21 play distinct roles in bone aging, a validated combination of senescence markers for different ages, sexes, and bone regions is still lacking.

Moreover, species differences between animal models and humans pose significant limitations for translational applications. Although estrogen deficiency-induced bone loss is well-understood in animals, many mechanisms, such as cytokine-mediated pathways, need further validation in humans due to study limitations (Weitzmann and Pacifici, 2006). Regarding the gut microbiome, significant changes are observed in postmenopausal rodents, but clinical studies in elderly women show minimal changes at the phylum level, suggesting that age-related comorbidities may impact the human gut microbiome more than menopause itself (Kverka and Stepan, 2024). Furthermore, the human gut is influenced by diet, medications, and lifestyle, which causes heterogeneity in clinical trials of interventions like probiotic supplementation (Fu et al., 2024). Despite these species differences, cellular senescence and its associated SASP in driving systemic inflammation are highly conserved across species (Jang et al., 2024). Thus, animal studies provide a mechanistic framework for the “skeletal aging triangle,” but their clinical application requires refinement through human tissue research and large-scale cohort data.

Finally, therapeutic strategies such as senescent cell clearance or estrogen replacement are still linked to risks like functional impairment and cancer, requiring more refined mechanistic understanding before clinical use.

### 6.3 Future research directions

Future investigations should focus on several key directions. First, advanced technologies such as single-cell sequencing and metabolomics can be leveraged to delineate the dynamic changes of the Skeletal Aging Triangle across different ages and sexes, thereby clarifying the relative contributions of each factor. Second, development of animal models that more closely mimic human skeletal aging—such as non-human primate models—will enhance the reliability of translational research. Third, further elucidation of the molecular mechanisms underlying the triangle’s interactions is needed to identify novel regulatory molecules and signaling pathways, which could serve as targets for next-generation therapies with improved specificity. Fourth, exploration of personalized intervention strategies based on an individual’s specific Skeletal Aging Triangle profile may allow precision medicine approaches to skeletal aging. Finally, large-scale, long-term clinical trials are required to evaluate both the efficacy and safety of combined intervention strategies, thereby paving the way for clinical translation. Collectively, these research efforts are expected to deepen our understanding of the Skeletal Aging Triangle and provide stronger theoretical foundations and practical guidance for the prevention and treatment of skeletal aging-related diseases.
